# Heterogeneous afferent arteriolopathy: a key concept for understanding blood pressure–dependent renal damage

**DOI:** 10.1038/s41440-024-01916-z

**Published:** 2024-10-08

**Authors:** Kentaro Kohagura, Ryo Zamami, Nanako Oshiro, Yuki Shinzato, Noriko Uesugi

**Affiliations:** 1 Dialysis Unit, University of the Ryukyus Hospital, Okinawa, Japan; 2 Department of Cardiovascular Medicine, Nephrology and Neurology Faculty of Medicine, University of the Ryukyus, Okinawa, Japan; 3 Department of Pathology, Fukuoka University School of Medicine, Fukuoka, Japan

**Keywords:** Antihypertensive therapy, Glomerular hypertension, Heterogeneous afferent arteriolopathy, Nephrosclerosis, Renal ischemia

## Abstract

Hypertension, aging, and other factors are associated with arteriosclerosis and arteriolosclerosis, primary morphological features of nephrosclerosis. Although such pathological changes are not invariably linked with renal decline but are prevalent across chronic kidney disease (CKD), understanding kidney damage progression is more pragmatic than precisely diagnosing nephrosclerosis itself. Hyalinosis and medial thickening of the afferent arteriole, along with intimal thickening of small arteries, can disrupt the autoregulatory system, jeopardizing glomerular perfusion pressure given systemic blood pressure (BP) fluctuations. Consequently, such vascular lesions cause glomerular damage by inducing glomerular hypertension and ischemia at the single nephron level. Thus, the interaction between systemic BP and afferent arteriolopathy markedly influences BP-dependent renal damage progression in nephrosclerosis. Both dilated and narrowed types of afferent arteriolopathy coexist throughout the kidney, with varying proportions among patients. Therefore, optimizing antihypertensive therapy to target either glomerular hypertension or ischemia is imperative. In recent years, clinical trials have indicated that combining renin–angiotensin system inhibitors (RASis) and sodium–glucose transporter 2 inhibitors (SGLT2is) is superior to using RASis alone in slowing renal function decline, despite comparable reductions in albuminuria. The superior efficacy of SGLT2is may arise from their beneficial effects on both glomerular hypertension and renal ischemia. A comprehensive understanding of the interaction between systemic BP and heterogeneous afferent arteriolopathy is pivotal for optimizing therapy and mitigating renal decline in patients with CKD of any etiology. Therefore, in this comprehensive review, we explore the role of afferent arteriolopathy in BP-dependent renal damage.

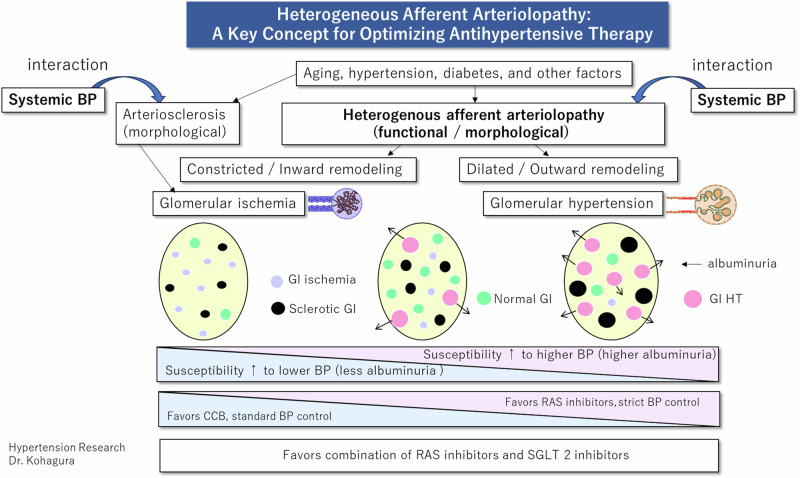

## Abstract (web summary)

Heterogeneous Afferent Arteriolopathy: A Key Concept for Optimizing Antihypertensive Therapy.

## Introduction

The global burden of hypertension is increasing [1], with major implications for renal health. Hypertension is a primary modifiable risk factor for deteriorating renal function and ranks among the leading causes of end-stage renal disease (ESRD) [2–5]. In Japan, nephrosclerosis has emerged as the second most common cause of ESRD in recent years [6]. Similarly, hypertension is the second leading cause of ESRD in the USA [7] and ranks third in China [8]. A nationwide survey in Japan revealed a linear increase in the initiation of hemodialysis therapy among elderly patients due to nephrosclerosis over the past decade [9]. The pathology of nephrosclerosis predominantly features arteriosclerosis and arteriolosclerosis [10–12]. Aging exhibits a linear association with these vascular lesions, observed across both younger healthy subjects [13] and those with chronic kidney disease (CKD) [14]. The interaction between systemic blood pressure and these vascular lesions may play a pivotal role in accelerating renal decline by inducing glomerular hypertension [15] and glomerular ischemia [16], recognized as common pathways in CKD development. Therefore, understanding the pathogenesis of progressive renal decline in the context of renal arteriosclerosis and arteriolosclerosis is crucial for formulating preventive strategies against CKD development from diverse etiology.

## Dilemma in defining nephrosclerosis and hypertensive nephrosclerosis

The term “nephrosclerosis” is often used interchangeably with hypertensive nephrosclerosis owing to shared pathological findings in patients with hypertension [10, 11, 17, 18]. Additionally, similar findings have been documented in elderly individuals [17]. Notably, indices of small artery intimal thickening increase progressively from younger to older age groups [13, 19]. Consequently, diagnosing nephrosclerosis typically relies on clinical observations, particularly in hypertensive patients with longstanding disease or in elderly individuals with renal dysfunction and minimal proteinuria, after excluding other primary renal diseases, such as glomerulonephritis [10, 16]. Given the frequent comorbidity of hypertension among the elderly [20], determining the causal factor for nephrosclerosis becomes challenging. Moreover, establishing precise diagnostic criteria proves difficult owing to similar findings associated with metabolic disorders, such as diabetes [21, 22], smoking [23], hypertension [18], and aging [13, 18, 19]. Previous studies have highlighted the diagnostic inaccuracies in identifying hypertensive nephrosclerosis in clinical practice [24].

Although delineating a distinct clinical entity for “(hypertensive) nephrosclerosis” remains elusive, it is indisputable that arteriosclerosis and arteriolosclerosis constitute primary pathologic features [12, 25, 26]. Kopp et al. advocated for adopting an etiologically neutral term, such as “arteriosclerosis,” to denote the disease rather than merely describing its pathology [27]. However, the vast number of hypertensive patients with renal impairment [28] renders renal biopsy impractical for diagnosing nephrosclerosis in elderly individuals with CKD and hypertensive patients. Additionally, vascular lesions akin to nephrosclerosis are observed in other renal diseases, including diabetic nephropathy [22] and glomerulonephritis [29], albeit to varying degrees. Determining the primary factors responsible for these vascular lesions remains challenging. Notably, morphological features consistent with nephrosclerosis do not invariably correlate with reduced renal function. For instance, elderly kidney transplant donors, generally considered free of CKD, exhibit a considerably higher prevalence of such morphological changes [13, 30]. Therefore, it is more pragmatic to assess how the pathogenic conditions associated with arteriosclerosis and arteriolosclerosis influence progressive renal function decline, rather than focusing on specific etiologies and precise diagnoses. Luke et al. suggested that the interaction between systemic blood pressure and arteriosclerosis and arteriolosclerosis may contribute to renal function decline [16]. In particular, morphological and functional alterations in the afferent arteriole (afferent arteriolopathy) contribute to glomerular hemodynamic abnormalities [31, 32]. In the following sections, we further explore the potential role of afferent arteriolopathy in blood pressure–dependent renal damage.

## Factors associated with arteriosclerosis and arteriolosclerosis

Numerous factors have been implicated in arteriolar hyalinosis, medial thickening, and small arterial intimal thickening, as summarized in Table 1. Additionally, their potential clinical relevance has been documented.

**Table 1:** Associated factors and clinical significance of arteriolosclerosis (hyalinosis and wall thickening) and arteriosclerosis (intimal thickening)

	Hyalinosis			Arteriolar wall thickening			Small artery intimal thickening		
Associated factor	Patients/subjects	Variables	Ref.	Patients	Variables	Ref.	Patients	Variables	Ref.
Aging	Donor, CKD (renal biopsy)	Age	[13, 14, 19, 35, 36]				Autopsy(general population), Donor,	Age	[35, 43]
Obesity				CKD (renal biopsy)*	Body mass index	[72]			
BP/Hypertension	Autopsy(general population), Autopsy(CVD)	SBP, DBP	[23, 35, 37–39, 45, 47]	Autopsy (general population)	Prehypertension	[37, 46]	Autopsy (general population)	Prehypertension Hypertension	[37]
	CKD (renal biopsy)	Prehypertension, hypertension		Nondiabetic CKD (renal biopsy)	Central BP				
	IgA nephropathy	BP variability,							
Glucose/Diabetes	CKD (renal biopsy), DKD,	Glucose, diabetes	[38, 40, 41]	DKD	Diabetes	[40]			
	Kidney allograft								
	Autopsy(CVD)								
Lipid	Autopsy(general population)	TC (men)	[35, 42]	CKD (renal biopsy)*	TG, HDL-C	[73, 74]			
	CKD (renal biopsy)	TG combined with C3		IgA N*	Adiponectin				
Uric acid	CKD (renal biopsy), Kidney allograft	Higher uric acid	[40, 50]	CKD (renal biopsy)	Higher uric acid	[40]A	Autopsy (general population)	Higher uric acid	[77]
Complement	CKD (renal biopsy)	C3	[51, 52]	Nondiabetic CKD (renal biopsy)	C3	[54]			
Cigarette smoking	IgA nephropathy	Smoking dose	[23, 42, 56]	IgA nephropathy*	Smoking & TG	[61]			[23, 78]
Alcohol	Autopsy(CVD)	Drinking history	[38]						
		(Negative correlation)							
RAS				CKD renal biopsy	UAGT	[66]			
				Hypertensive patients	RAS inhibitor	[69]			
**Clinical significance**	**Patients**	**Outcomes**	**Ref**.	**Patients**	**Outcomes**	**Ref**.	**Patients**	**Outcomes**	**Ref**.
Proteinuria, UACR	CKD (renal biopsy), DKD	Changes in UACR	[79, 80]						
	IgA nephropathy	Macroalbuminuria							
	kidney allograft								
Renal progression	DKD, IgA nephropathy	eGFR < 60 ml/min/1.73 m^2^	[80–85, 87]	IgA nephropathy*	50% eGFR decline, ESRD	[88]	IgA nephropathy,	30–50% eGFR decline, ESRD	[83, 97]
		25–50% eGFR decline					DKD,		
ESRD	DKD, IgA nephropathy, kidney allograft	ESRD	[82–86]				DKD	ESRD, Death	[98]

*BP* blood pressure, *UACR* urinary albumin to creatinine ratio, *ESRD* end-stage renal disease, *CKC* choric kidney disease, *DKD* diabetic kidney disease, *SBP* systolic blood pressure, *DBP* diastolic blood pressure, *TC* total cholesterol, *TG* triglyceride, *HDL-C* high-density lipoprotein cholesterol, *C3* complement C3, *UAGT* urinary angiotensinogen, *RAS* renin–angiotensin system

* Indicates the study examined the Hyalinosis & wall thickening

## Renal arteriolar hyalinosis

Hyalinosis constitutes a major morphological feature in arteriolosclerosis, characterized by the deposition of pink amorphous material primarily within intimal spaces [33]. Studies have indicated that these deposits mainly consist of complement C3 [34]. Although the mechanisms underlying hyaline deposit formation remain unclear, some substances in the blood infiltrate the arteriolar wall due to endothelial damage [33]. Hyalinosis has been associated with classical risk factors, such as aging [13, 14, 19, 35, 36], blood pressure/hypertension [23, 35, 37–39], glucose/diabetes [38, 40, 41], lipid abnormalities [35], and cigarette smoking [23, 42].

Prevalence data have revealed a linear increase in morphological findings of nephrosclerosis and small arterial intimal thickening from the 20 s to 80 s age groups in healthy adult renal transplant donors [13]. Similarly, arteriosclerosis has been observed in children, with its severity increasing even before adolescence [19, 36]. Given that hypertension and diabetes are rare in younger individuals, these results suggest that aging itself is an important risk factor for the pathologic findings. In an autopsy study, the interaction between aging and hypertension was linked to arteriolar hyalinosis [43]. Additionally, nonclassical risk factors, such as oxidative stress, inflammation, and uremic toxins, are considered contributors to arteriosclerosis in patients with CKD [44]. Therefore, the additive effect of classical and nonclassical risk factors may exacerbate the aging-associated risk for arteriolosclerosis. In patients undergoing renal biopsy, the prevalence of arteriolar hyalinosis and small arterial intimal thickening tends to increase from 20 s and markedly rises from 30 s compared with the teenage years [14]. Conversely, the association of aging with the wall-to-lumen ratio, an indicator of medial remodeling, is weak.

Regarding blood pressure, both systolic [23, 35] and diastolic [38] blood pressure have been linked to arteriolar hyalinosis. Furthermore, masked hypertension and sustained hypertension are associated with arteriolar hyalinosis in patients with chronic glomerular diseases [45]. Additionally, pulse wave velocity [46] and blood pressure variability are correlated with hyalinosis [47].

Arteriolar hyalinosis is often observed in patients with diabetic nephropathy [22]. A previous study examining nephrectomy specimens revealed moderate arteriolar hyalinosis in approximately 90% of patients with diabetes, irrespective of albuminuria status [48]. Diabetes or glucose abnormalities have also been associated with arteriolar hyalinosis in autopsy [38] and renal biopsy studies [40]. Baseline diabetes has been linked to the development of arteriolar hyalinosis in renal transplant recipients [41]. Similarly, hypercholesterolemia [35] and indices of insulin resistance [39] have been associated with renal arteriolar hyalinosis.

In a rat model of hyperuricemia, uric acid was implicated in inducing arteriolar lesions, possibly contributing to hypertension and renal dysfunction [49]. Higher levels of uric acid are associated with arteriolar hyalinosis in patients undergoing renal biopsy [40]. Similarly, an association between arteriolar hyalinosis and uric acid was demonstrated in renal allografts [50].

Complement C3 deposition and activation in arteriolar lesions have been observed in arterionephrosclerosis [51] and IgA nephropathy [52]. C3-induced increases in vascular permeability accompanied by neutrophil migration have also been reported [53]. Although C3 is markedly increased through the formation of immune complexes, it is also a known adipocytokine, and serum C3 levels are significantly positively correlated with triglyceride levels [54], suggesting that the serum C3–triglyceride interaction may contribute to the progression of metabolic syndrome by stimulating phenotypic changes in visceral adipocytes. When accompanied by elevated serum C3 levels, hypertriglyceridemia is strongly associated with arteriolar hyalinosis in patients with CKD [55].

Smoking has also been suggested as a factor associated with arteriolar hyalinosis [42]. For instance, a previous study showed that prominent arteriolar hyalinosis was observed in individuals with a history of cigarette smoking without hypertension and diabetes [56].

In addition to classical risk factors, previous reports have demonstrated associations between arteriolar hyalinosis and various factors, including Klotho deficiency [57] and N-terminal pro-brain natriuretic peptide levels [58]. A study involving single-cell transcriptomic data analysis suggested associations between renal arteriolar hyalinosis and transforming growth factor beta (TGF-β)/bone morphogenetic protein/vascular endothelial growth factor signaling [59]. Common mechanisms may be involved in the pathogenesis of renal arteriolar hyalinosis regardless of etiology, as TGF-β activation has also been associated with calcineurin inhibitor-induced afferent arteriolar hyalinosis [60].

## Factors associated with renal arteriolar medial thickening

Various factors have been associated with renal arteriolar medial thickening. Prehypertension and hypertension were linked to arteriolosclerosis in the general population in an autopsy study [37]. Additionally, central blood pressure may correlate with arteriolar wall thickening in young to middle-aged patients with nondiabetic kidney disease and preserved renal function [46]. We previously found that higher levels of uric acid and diabetes were associated with arteriolar wall thickening in patients with CKD undergoing renal biopsy [40]. Moreover, smoking combined with elevated uric acid levels was associated with a greater arteriole wall-to-lumen ratio [61].

In genetically hypertensive rats, renin–angiotensin system (RAS) inhibitors prevent arteriolar remodeling [62, 63] In Dahl salt–sensitive rats, RAS activation contributes to hypertension development by inducing renal arteriolar wall thickening, a pathogenic process inhibited by RAS inhibitors [64]. Additionally, a high-salt diet can initiate such processes by inducing arteriolar medial thickening [65]. We previously reported a significant association between urinary angiotensinogen, a potential intrarenal RAS indicator, and the arteriolar wall-to-lumen ratio in patients with CKD not treated with RAS inhibitors [66]. These findings suggest a potential link between intrarenal RAS and medial smooth muscle proliferation. Contrary to these findings, studies have shown that RAS inhibition induces smooth muscle layer proliferation in the afferent arteriole of hypertensive patients and animal models of hypertension [67–69]. However, Nagai et al. reported that renin inhibitors did not produce such vascular lesions [70]. Moreover, RAS inhibition, either through medication or genetic knockout of angiotensinogen, may cause marked smooth muscle cell proliferation by inducing phenotypic changes toward the synthetic type in renin-producing cells [71]. These findings imply that RAS inhibitor treatment may lead to glomerular hypoperfusion and subsequent tubular ischemia.

Obesity [72] and certain lipid abnormalities [73, 74] have also been associated with arteriolosclerosis, characterized by arteriolar wall thickening and hyalinosis.

Additionally, complement C3 deposition in arterioles has been correlated with indices of wall thickening and hyalinosis in patients with biopsy-confirmed arterionephrosclerosis [54]. One study suggested that phenotypic changes in profibrotic secretory vascular smooth muscle cells may mediate arteriolar fibrosis [75]. Another study indicated that increased levels of MMP-9, which plays a pivotal role in hypertension-induced kidney microvascular remodeling [76], promote the phenotypic transformation of afferent arterioles, resulting in the loss of myogenic constriction and hypertensive nephropathy [76].

## Factors associated with small arterial intimal thickening

Autopsy studies [35, 43], and examinations of patients with CKD who underwent renal biopsy [14] have demonstrated associations between small arterial intimal thickening and various factors. Prehypertension and hypertension were associated with a higher wall-to-lumen ratio of renal small arteries in an autopsy study [37], and in another autopsy study, elevated uric acid levels were linked to arterial intimal thickening [77]. Furthermore, smoking was associated with intimal thickening in renal biopsy studies [23, 78]. Animal studies have indicated that a high-salt diet induces medial thickening of renal arterioles and subsequent hypertension via enhanced salt sensitivity [65].

## Clinical importance of arteriosclerosis and arteriolosclerosis

### Hyalinosis and renal outcomes

Renal arteriolar hyalinosis has been associated with adverse renal outcomes across various clinical settings. In patients with diabetes, hyalinosis was linked to the development of albuminuria [79, 80], rapid decline in estimated glomerular filtration rate (eGFR) [81], incidental CKD [80], and ESRD [82]. Similarly, in patients with IgA nephropathy, hyalinosis was associated with substantial declines in eGFR (30% or 50%) and progression to ESRD [83–85]. In renal transplant recipients, hyalinosis was correlated with long-term graft function and graft loss [86, 87]. Furthermore, arteriolar damage, defined by the presence of arteriolar hyalinosis and medial wall thickening, was associated with poor renal survival in patients with lupus nephritis [88].

## Mechanisms underlying the association between hyalinosis and poor renal outcomes

### Hyalinosis and susceptibility to hypertensive renal damage

In some studies, hyalinosis exerted no significant effect on renal outcomes in patients with hypertensive nephrosclerosis [89] or diabetic nephropathy [82, 90, 91]. These findings raise questions regarding the independent role of hyalinosis in CKD progression. However, the interactive effect of hyalinosis and systemic blood pressure on glomerular hemodynamics may play a crucial role in such progression.

Hill et al. indicated that hyalinosis may serve as a morphologic marker of disrupted autoregulation in the afferent arteriole, based on findings of dilated afferent arteriole diameter with hyalinosis and enlargement in connected glomeruli [31]. We previously investigated the potential role of hyalinosis in hypertensive glomerular damage, examining a patient with non-nephrotic CKD [92]. We found that proteinuria levels increased with increasing systolic blood pressure in patients with arteriolar hyalinosis but not in those without this condition. Moreover, the combination of hypertension and arteriolar hyalinosis was significantly associated with higher levels of proteinuria independently. These findings support the notion that hyalinosis is associated with a disrupted autoregulation system in the afferent arteriole, leading to CKD progression due to enhanced susceptibility to hypertensive renal damage. In the same study [92], subgroup analysis demonstrated a greater decline in eGFR with increasing systolic blood pressure, a relationship augmented by the presence of renal arteriolar hyalinosis in patients with IgA nephropathy. This finding aligns with Hill’s suggestion that hyalinotic deposition may impair smooth muscle contraction in the afferent arteriole, thereby disrupting autoregulation systems [31]. Consistent with this hypothesis, a cross-sectional study conducted in patients with non-nephrotic CKD showed a significant positive correlation between systolic blood pressure and proteinuria in individuals with higher uric acid levels associated with renal arteriolar hyalinosis, whereas this trend was not observed in individuals without this condition [93].

## Hyalinosis and ischemic renal damage

Severe hyalinosis accompanied by narrowing of the afferent arteriole lumen has been suggested to be associated with ischemic damage in connected glomeruli [31]. The presence of arteriolar hyalinosis represents an additional risk for ischemic injury in renal transplants [94]. Moreover, hyalinosis may serve as a potential surrogate marker of reduced interstitial blood flow and hypoxia in patients with glomerulonephritis [95]. In addition, an observed association between advanced arteriolar hyalinosis and poor renal outcomes has been associated with the induction of collapsing glomerular damage accompanied by ischemic podocyte injury [96].

## Arteriolar medial thickening, small arterial intimal thickening, and renal outcomes

There is no clear evidence suggesting that arteriolar medical thickening is responsible for CKD progression. However, small arterial intimal thickening has been associated with an increased risk of composite renal outcomes (30% decline in eGFR or renal replacement therapy) in patients with diabetic nephropathy [97]. Severe arterial intimal thickening is associated with worse renal outcomes in patients with diabetic nephropathy [98] and those with IgA nephropathy [83]. Moreover, arteriolosclerosis lesions involving arterial intimal fibrosis and arteriolar hyalinosis are correlated with composite renal outcomes (a 50% eGFR decline and ESRD) in patients with IgA nephropathy [29].

## Mechanisms underlying the effects of small arterial intimal thickening on renal outcomes

The narrowing of the lumen due to intimal thickening of small arteries can lead to renal ischemia, especially during reductions in blood pressure in various conditions, including dehydration.

## Susceptibility to blood pressure–dependent renal damage

Biddani and Griffin provided an important concept for understanding the underlying mechanisms involved in susceptibility to blood pressure–dependent renal damage [99]. For example, renal injury is unlikely to occur in patients with essential hypertension unless their blood pressure is elevated, as observed in malignant hypertension [99]. An increase in vascular resistance in the afferent arteriole has been observed in patients with essential hypertension [100], and narrowing of the afferent arteriole may precede the development of hypertension, likely due to elevated peripheral vascular resistance [101]. Conversely, increased vascular resistance in the afferent arteriole may protect glomeruli by preventing high systemic blood pressure from being transmitted directly to the glomerulus. The slow progression of renal injury generally observed in essential hypertensive patients may be attributed to ischemia-driven pathology via narrowing of the arteriole lumen and small artery [102]. In contrast, even slight increases in systemic blood pressure can cause glomerular damage in patients with other CKDs, such as diabetic nephropathy and advanced CKD, due to glomerular hypertension induced by autoregulatory mechanism disruption in the afferent arteriole [102], with the autoregulatory mechanisms including myogenic and tubuloglomerular feedback [32]. The linear increase in risk observed between blood pressure and the development of kidney injury may be attributable to impaired autoregulation systems [99].

Arteriolar hyalinosis may be responsible for impaired autoregulation of the afferent arteriole as well as augmented susceptibility to hypertensive renal damage [92]. In a study of biopsy-confirmed nephrosclerosis, patients with a body mass index ≥25 kg/m^2^ exhibited a significant positive correlation between systolic blood pressure and albuminuria [103]. Kinkade-Smith suggested that obesity and metabolic syndrome are more strongly associated with secondary segmental glomerulosclerosis than hypertension during renal failure attributed to hypertensive nephrosclerosis [104]. Given that hyperinsulinemia is a factor associated with autoregulation system disruption in the afferent arteriole [105], obesity may enhance susceptibility to hypertensive renal damage in patients with hypertension.

African Americans are at increased risk of developing renal impairment, with the condition exhibiting distinct racial differences [106]. For instance, apolipoprotein L1 (*APOL1*) gene renal-risk variants are strongly associated with nondiabetic glomerulosclerosis and all cases of ESRD in African Americans. Freedman reported the significance of *APOL1*‑associated glomerulosclerosis as a distinct clinical entity characterized by solidification rather than obsolescence in arteriolar nephrosclerosis [12]. Another study demonstrated the localization of APOL1 in the arteriolar endothelium of diseased kidney sections, including focal glomerulosclerosis in African Americans [107]. The relationship between blood pressure and glomerular volume is augmented in African Americans compared with their white counterparts [18]. These findings imply that APOL1-associated changes in the afferent arteriole may be linked to enhanced susceptibility to hypertensive glomerular damage via disruption of autoregulation systems.

## Heterogeneous afferent arteriolopathy and heterogeneous glomerular morphology

**Fig. 1:**
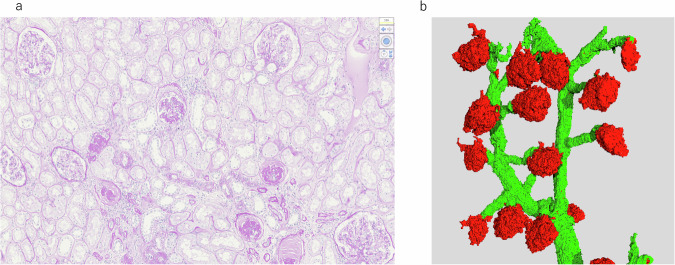
**a** Renal specimen from a nephrectomy kidney with a normal lesion due to renal cell carcinoma in patients with nephrosclerosis. Glomerular sizes varied markedly among glomeruli. **b** Three-dimensional nephron tree. Glomerular volumes varied substantially among glomeruli

In patients with nephrosclerosis, glomerular sizes and volumes varied widely among glomeruli throughout the kidney (Fig. 1). Laragh et al. indicated the existence of two functionally abnormal nephron populations in the whole kidney of individuals with essential hypertension: ischemic nephrons and adapting hyperfiltrating normal nephrons [108]. Ischemic nephrons are attributed to narrowed afferent arterioles. Tracy et al. revealed that focal intimal fibroplasia associated with blood pressure may be responsible for the heterogeneous pattern of ischemic glomeruli [109]. Hill et al. showed a wide distribution in both the afferent arteriole’s lumen diameter and glomerular size in elderly individuals [31]. The rate of swollen glomeruli accompanied by a large lumen diameter in the afferent arteriole is comparable to that of smaller glomeruli accompanied by a small lumen diameter. Moreover, in patients with hypertension, the rate of smaller glomeruli accompanied by a lower lumen diameter in the afferent arteriole is relatively high. Elevated perfusion pressure leads to an increase in glomerular volume [110]. Accordingly, swollen glomeruli are considered to reflect glomerular hypertension. In an autopsy study, glomerular volume in the juxtamedullary cortex tended to be larger than that in the superficial cortex, accompanied by a significantly higher arteriolar hyalinosis score in diabetic patients [111]. Therefore, it has been suggested that glomeruli suffering from glomerular hypertension in association with arteriolar hyalinosis exist heterogeneously in the kidney. Previous research involving models of aging and hypertension revealed that juxtamedullary nephrons are larger than superficial nephrons and that a greater proportion of proteinuria originates from the former [112]. A study involving a diabetic model rat demonstrated a heterogeneous pattern of absorbed albumin in the tubules, with the rate of such findings increasing with albuminuria [113]. These results may account for heterogeneous glomerular hypertension. In studies examining normal lesions in kidneys with renal cell carcinoma, swollen glomeruli were found to coexist with collapsed glomeruli [31, 114]. Given that collapsed glomeruli occur due to hypoperfusion, these findings suggest that glomerular hypertension and ischemia coexist throughout the kidney. Collectively, the abovementioned findings suggest that heterogeneity in afferent arteriolopathy, consisting of dilated and narrowed lumens, and in small arterial intimal thickening with narrowed lumens, may result in a heterogeneous distribution of hypertension-affected and ischemic glomeruli.

## Heterogeneous blood pressure–dependent renal damage

**Fig. 2:**
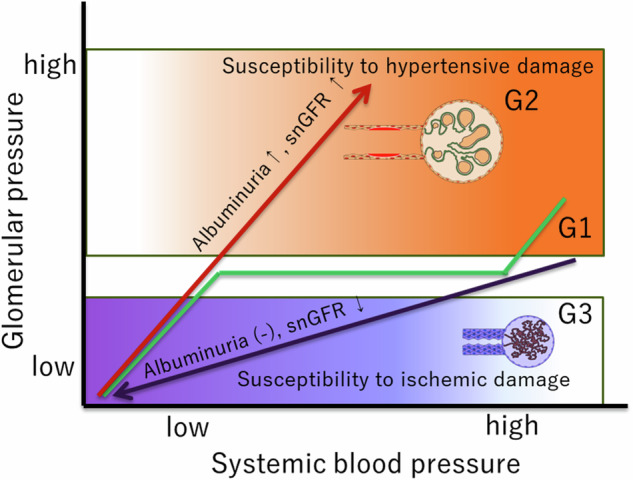
Conceptual relationship between systolic blood pressure and glomerular pressure based on types of afferent arteriolopathy and glomerular hemodynamic abnormalities, G1, normal afferent arteriole with regular glomerular hemodynamics: glomerular blood pressure is maintained across a wide range of systemic blood pressure levels; G2, dilated/outward remodeling type of afferent arteriolopathy with glomerular hypertension: glomerular blood pressure increases linearly with systemic blood pressure, leading to glomerular hypertension even within the normal range of systemic blood pressure; G3, narrowed/inward remodeling type of afferent arteriolopathy with glomerular ischemia: glomerular pressure decreases linearly with systemic blood pressure, resulting in glomerular ischemia even within the normal range of systemic blood pressure. snGFR single nephron glomerular filtration rate

**Fig. 3:**
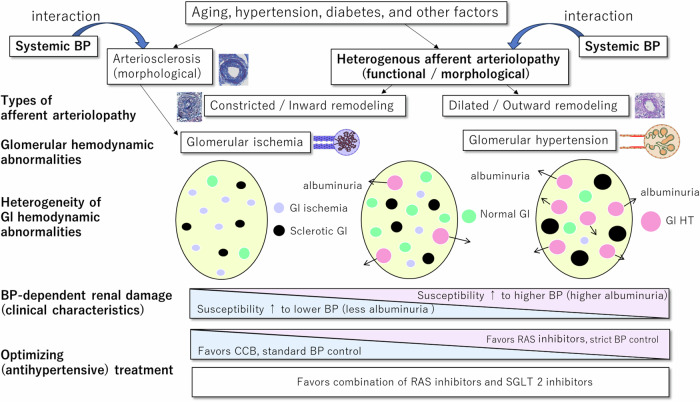
Associations of heterogeneous afferent arteriolopathy and small arterial intimal thickening with heterogeneous glomerular hemodynamic abnormalities. A higher rate of glomeruli connected with dilated lumen, characterized by increased proteinuria levels, is associated with heightened susceptibility to high blood pressure–dependent renal damage. Conversely, a higher rate of glomeruli connected with a narrowed lumen, characterized by less proteinuria, is associated with increased susceptibility to low blood pressure–dependent renal damage. BP blood pressure, Gl glomerulus or glomerular, RAS renin–angiotensin inhibitor, CCB calcium channel blocker, SGLT 2 sodium–glucose cotransporter 2

At the single nephron level, the autoregulatory system in the afferent arteriole maintains stable glomerular pressure despite fluctuations in systemic blood pressure. However, afferent arteriolopathy can disrupt this balance, leading to glomerular hypertension and/or ischemia. Disrupted autoregulation in the afferent arteriole exacerbates susceptibility to hypertensive renal damage; therefore, under conditions with dilated afferent arteriolopathy (Fig. 2, G2), even slight increases in systemic blood pressure can directly transmit pressure to the glomerulus, causing glomerular hypertension. Conversely, under conditions with narrowed afferent arteriolopathy (Fig. 2, G3), even normal systemic blood pressure levels can result in ischemic glomerular damage due to hypoperfusion. Given that both types of afferent arteriolopathy, namely dilated and narrowed, coexist in the kidney at varying rates, the overall impact of these vascular lesions depends on the interaction between systemic blood pressure and the prevalence of each afferent arteriolopathy type (Fig. 3). For example, even systolic blood pressure levels of 130 mmHg can induce glomerular damage in individuals with a higher prevalence of dilated afferent arteriolopathy, whereas systolic blood pressure levels of 120 mmHg can cause ischemic glomerular damage, a scenario often referred to as “normotensive ischemic acute renal failure” [115].

In clinical settings, proteinuria serves as a marker indicating the presence of glomerular hypertension resulting from dilated afferent arteriolopathy. Furthermore, the relationship between blood pressure levels and renal decline is augmented in patients with proteinuria, suggesting that the condition may represent a practical marker indicating increased susceptibility to blood pressure–dependent renal function decline [116]. In a previous animal study, albuminuria levels correlated with the distribution of nephrons leaking albuminuria [113]. Therefore, higher levels of albuminuria may reflect a higher prevalence of glomeruli experiencing glomerular hypertension.

## Therapeutic strategies based on glomerular hypertension and ischemia proportions

Optimizing target blood pressure levels and selecting the appropriate antihypertensive drugs are crucial for preventing renal decline, considering their potential impact on heterogeneous glomerular hypertension and ischemia (Fig. 3). Insightful findings from the African-American Study of Kidney Disease and Hypertension (AASK) shed light on this matter. The AASK study, conducted among African Americans with nephrosclerosis, revealed that in all patients, RAS inhibitors were superior to calcium channel blockers or beta-blockers, and tight blood pressure control was comparable to standard blood pressure control in preventing a composite renal endpoint, including ESRD [117]. However, the results differed in terms of effects on eGFR decline between patients with ≥0.22 and <0.22 g/gCr of proteinuria. In the former group, strict blood pressure control and RAS inhibitor treatment were favorable for preventing eGFR decline [117]. A previous experimental study involving a remnant kidney model showed that calcium channel blockers increased hypertension-induced glomerular damage compared with the control and RAS inhibitors [118]. Conversely, in patients with proteinuria at <0.22 g/gCr, calcium channel blockers were superior to RAS inhibitors in preventing eGFR decline during the study period, and tight blood pressure control was comparable to standard blood pressure control in its outcome [117].

In patients with CKD enrolled in the AASK or Modification of Diet in Renal Disease studies, strict blood pressure control was beneficial in reducing the risk of ESRD in a subgroup with proteinuria at ≥0.44 g/gCr. These findings align with the notion that strict blood pressure control may be more favorable for preventing declining renal function in patients with higher proteinuria, where a higher rate of glomeruli experiencing glomerular hypertension is assumed. Conversely, hypertensive patients with proteinuria at <0.22 g/gCr may exhibit a higher rate of ischemic glomeruli. Strict blood pressure control increased the risk of incidental decline in eGFR to <60 ml/min/1.73 m^2^ in the Systolic Blood Pressure Intervention Trial (SPRINT), conducted mainly in elderly patients with hypertension [119]. Given that baseline albuminuria was extremely low in SPRINT, it was suggested that ischemic glomeruli may be damaged via induced hypoperfusion after strict blood pressure control. Theoretically, even at a blood pressure of 120/80 mmHg, ischemic glomeruli caused by a narrowed lumen in the afferent arteriole can cause glomerular hypoperfusion, resulting in normotensive ischemic acute kidney injury [115]. This may be caused by damage to potentially ischemic glomeruli due to stricter blood pressure control.

In a renovascular hypertension model, RAS inhibitors were found to reduce the eGFR of the ischemic kidney [120]. In patients with bilateral renal artery stenosis, RAS inhibitors caused acute kidney injury [121]. An animal study demonstrated that the renal function of a single kidney with unilateral renal vascular stenosis was reduced by RAS inhibitors, whereas function remained unimpaired in the other kidney lacking renal artery stenosis [120]. Given that a similar ischemic condition can be induced by globally distributed severe arteriosclerosis and/or arteriolosclerosis with narrowed lumen, RAS inhibitors may interfere with compensatory vasoconstriction of the efferent arteriole by angiotensin II [115]. In a meta-analysis of randomized trials, RAS inhibitors reduced renal failure events compared with the placebo or agents in a proteinuria-positive group [122]. However, they showed no significant effect on the risk of renal failure in the proteinuria-negative group, despite reducing microalbuminuria levels. This observation suggests that there may be a factor associated with RAS inhibitors that offsets the benefits of correcting glomerular hypertension.

Given the heterogeneous distribution of glomerular hypertension and ischemic glomeruli among many patients, the clinical benefits of RAS inhibitors are　contingent on striking a balance between their favorable and unfavorable effects on glomerular hypertension and ischemic glomeruli, respectively (Fig. 3). When patients exhibit a similar proportion of glomerular hemodynamic abnormalities, the impact of RAS inhibitors on glomerular hypertension and ischemic glomeruli tends to be comparable, resulting in a neutral effect on the risk of renal failure.

Overall, the use of stringent antihypertensive therapy and RAS inhibitors presents a potential dilemma with both advantages and disadvantages for many patients with heterogeneous glomerular hypertension and ischemia. Ideally, a treatment that effectively corrects glomerular hypertension without exacerbating ischemia, or even ameliorates ischemia, would be optimal. In recent years, clinical trials have suggested potential solutions to this dilemma. For instance, in the EMPA-KIDNEY trial (Study of Heart and Kidney Protection with Empagliflozin), among patients with normal albuminuria, the addition of sodium–glucose transporter 2 inhibitor (SGLT2i), empagliflozin to the standard treatment with maximally tolerated RAS inhibitors resulted in significantly slower changes in eGFR compared with the standard treatment alone, regardless of baseline albuminuria levels [123]. Individuals with normal albuminuria in the control group treated with a RAS inhibitor alone showed a mild decrease in eGFR [123], suggesting that factors other than glomerular hypertension controlled by RAS inhibitors may contribute to eGFR decline.

Renal ischemia, a common pathway to renal decline [124], is one of the plausible contributors to progressive eGFR decline in patients with CKD but without albuminuria. The Dapagliflozin and Prevention of Adverse Outcomes in Chronic Kidney Disease (DAPA-CKD) trial, conducted among diabetic and nondiabetic patients with CKD and overt albuminuria, despite administration of maximally tolerated doses of RAS inhibitors [125], revealed that the addition of dapagliflozin to RAS inhibitors led to a decrease in albuminuria, which was associated with a reduction in eGFR at two weeks after initiation [126]. Moreover, a greater reduction in albuminuria at two weeks was correlated with a slower decline in eGFR during the chronic phase [126]. These results suggest that the decrease in renal events may primarily result from the improvement in glomerular hypertension. Furthermore, subanalysis of the DAPA-CKD trial revealed that the rate of decline in eGFR during the chronic phase was two-to-three times slower in the DAPA group (dapagliflozin + RAS inhibitor) compared with the control group (RAS inhibitor), despite similar reductions in albuminuria between the groups [126]. For instance, if a 40% decrease in albuminuria was achieved at two weeks, the control group exhibited a delta eGFR value of approximately −3 ml/min/1.73 m^2^, whereas the DAPA group showed a delta eGFR value of approximately −1.2 ml/min/1.73 m^2^ [126]. These findings suggest that factors other than improving glomerular hypertension may contribute to the renoprotective effects of dapagliflozin.

Previous studies have shown that SGLT2is improves oxygenation by reducing the activity of Na-K ATPase, a major contributor to oxygen consumption in tubules [127, 128]. A meta-analysis of clinical trials revealed that SGLT2is increases hemoglobin levels [129]. Additionally, SGLT2is may enhance peritubular capillary density through the vascular endothelial growth factor–dependent pathway [130]. These findings suggest that SGLT2is can improve renal hypoxia by alleviating the imbalance between oxygen consumption and supply. Furthermore, a recent clinical study employed functional magnetic resonance imaging in patients with newly diagnosed type 2 diabetes mellitus and confirmed that SGLT2is improves renal oxygenation indices [131].

Given the heterogeneous distribution of glomerular hypertension and ischemia, RAS inhibitors may ameliorate glomerular hypertension while potentially worsening glomerular ischemia. In ischemic glomeruli, the increased delivery of sodium and chloride to macula densa cells by SGLT2is is unlikely to restore or surpass the delivery levels observed at normal glomerular filtration rates. Therefore, SGLT2is may not further reduce the glomerular filtration rate in ischemic glomeruli via tubuloglomerular feedback mechanisms. In the DAPA group (dapagliflozin + RAS inhibitor), dapagliflozin may counteract or neutralize the detrimental effect of RAS inhibitors on ischemic glomeruli. Notably, the DAPA-CKD trial revealed a beneficial effect of dapagliflozin in hypertensive nephrosclerosis/ischemic nephropathy. Furthermore, a previous study showed that the BP-lowering effects of dapagliflozin are more pronounced in diabetic patients with high-sodium intake, suggesting that SGLT2is may also aid in blood pressure control in patients with CKD [132]

## Summary and future implications

Heterogeneous afferent arteriolopathy is believed to be prevalent, especially among middle-aged to elderly patients with comorbidities, such as hypertension, and diabetes. Interaction between this afferent arteriolopathy and systemic blood pressure likely plays a pivotal role in the decline of renal function through exacerbation of both glomerular hypertension and ischemia, which are common pathways leading to CKD. Future studies should aim to develop individually optimized therapeutic strategies considering the heterogeneity of afferent arteriolopathy in different etiologies of CKD.
